# Editorial: Coping With Pandemic: Families Engagement and Early Parental Intervention to Support Child Development During and After the COVID-19 Outbreak

**DOI:** 10.3389/fpsyg.2022.968945

**Published:** 2022-07-12

**Authors:** Eleonora Mascheroni, Barbara Kalmanson, Mark S. Innocenti, Rosario Montirosso

**Affiliations:** ^1^0–3 Center for the At-Risk Infant, Scientific Institute IRCCS Eugenio Medea, Bosisio Parini, Italy; ^2^KidsAttuned.org, Fielding Graduate University, Santa Barbara, CA, United States; ^3^Institute for Disability Research, Policy and Practice, Utah State University, Logan, UT, United States

**Keywords:** parenting, parenting intervention, COVID−19, child wellbeing, resilience

The public health emergency due to Coronavirus disease 2019 (COVID-19) that started more than 2 years ago required significant measures to ensure infection control that resulted in public health, social and economic challenges worldwide. While social-distancing, quarantine, and isolation measures were proved to be effective to reduce community-based transmission, their psychological cost is still increasingly evident (Prati and Mancini, 2021). The uncertainty and lack of predictability associated with the pandemic in these recent years has been a highly stressful and traumatic experience for children, adolescents and their families (Alonzi et al., 2021; Panchal et al., 2021).

A growing body of research is revealing the presence of both short-term and mid-term detrimental consequences on children's mental health and psychological adjustment, suggesting that these may continue long-term for many children. This scenario is exacerbated by the stress experienced by parents, potentially affecting their ability to provide consistent care and support that may negatively impact the parent-child relationship. In these past 2 years, many parents had to care for children while working from home, supervise home-based schooling, and deal with economic uncertainty. Demands that may be even greater for parents who must care for children with special needs or disabilities (Montirosso et al., 2021).

Since parenting is a critical factor in early child development, for this special issue we called for research papers that questioned what factors may amplify or mitigate the negative effects of COVID-19 on children and their parents. In addition, we wanted articles that provided evidence of effective parenting interventions that would support the child as well as the family system in dealing with the challenges associated with the COVID-19 pandemic. We identified four themes in the published articles.

The first theme, stress begets stress, focuses on risk factors related to the caregiving and family environment that can amplify the effect of COVID-19 on child psychological adjustment. Radanović et al. reported that both parent's fear and children's exposure to negative pandemic information were associated with an increase in the children's fear of COVID-19 (this finding was supported by de Vet et al.). Parents scoring higher on separation anxiety and fear of COVID-19 experienced more distress which was associated with higher children distress once they re-entered child care services. These results were more evident in younger children. Parental distress was not only associated to a higher fear of COVID-19 but also to other contextual factors. Thibodeau-Nielsen et al. found that economic hardships were related to increased caregiver stress, which was associated with children's emotional distress and poorer self-regulation. However, the negative association between parents' stress and children's emotional difficulties was moderated by children's ability to engage in pandemic-related play. Finally, de Vet et al. focused on the impact of COVID-19 on child wellbeing after the lockdown, when the Child Care Services reopened. Younger children and children with parents scoring higher on separation anxiety experienced more distress after the reopening. These studies highlight the need to study moderators of stress during high stress events both during and after the events.

Another theme, stress multipliers, is reflected in the studies that analyzed subjects already in at-risk situations that may potentially amplify the stress effects of COVID-19. The psychological adjustment associated with the pandemic can be particularly difficult for all individuals, research suggests that the psychological impact of COVID-19 may be more severe for some at risk populations (Boyraz and Legros, 2020; Chaix et al., 2020; Stefana et al., 2020). Manuela et al. observed an increase of depression symptoms in mothers of extremely preterm children (born before 32 weeks gestation) hospitalized in Neonatal Intensive Care Unit (NICU) during the COVID-19 pandemic, which were associated with less postnatal attachment and higher maternal stress. He et al. focused on economically vulnerable families. The difficulties (lay-offs, reduced work, etc.) faced by low-income families during the pandemic put them at higher risk for negative short and long-term consequences. In these families, parents reported increased financial strain and more mental health difficulties, especially for fathers, during the pandemic. Moreover, children exhibited more behavior problems compared to before the pandemic. These studies emphasize the need for research on approaches to reduce stress in vulnerable groups during high-stress times.

While some research focused on risk factors and at-risk condition that can negatively affect the wellbeing of children and parents, others have focused on protective factors. The third theme, promoting positive family behaviors, focused on promoting resilience and teaching positive behavior among the family system. Both Johnson et al. and Mariani Wigley et al. observed that parent's ability to teach children resilient behavior, to enhance acts of kindness and to develop trusting relationships can improve child adjustment during the pandemic. The potential contributions of family resilience during the COVID-19 pandemic to parents and children had more positive outcomes for low-income families (He et al.). Baggett et al. reported low-income and depressed mothers, at high risk for poor developmental outcomes, were supported by an internet-based parenting intervention with virtual coaching. Evidence-based remote coaching interventions were reported as crucial during the pandemic, especially for at-risk families. Preliminary findings from an ongoing randomized controlled trial study showed rates of successful progression into intervention that were at least as favorable as those reported in routine studies of home visiting intervention programs outside of pandemic. These studies demonstrate approaches to maintain positive family behaviors during times of high stress.

Our fourth theme focuses on child services (care and early intervention) during the pandemic and some very interesting findings resulted. As a consequence of the COVID-19 outbreak child care services all over the world were temporarily closed to minimize the spread of the virus. However, in most of the cases these organizations worked hard to continue serving children and their families during the COVID-19 lockdown using online applications. These new service approaches seemed to have a positive impact on families. Nossa et al. reported that online, organized activities decreased the sense of loneliness and boredom for children and acted as a crucial support for parents. For children with special needs, Vilaseca et al. observed that the virtual provision of Early Intervention services was positively perceived by parents, especially for parents who took care of their child during the day and used online tools before the lockdown. Telematics (virtual) intervention during COVID-19 became an opportunity for practitioners to encourage families' participation, promoting an effective model of family-centered care. These studies demonstrate thoughtful and effective methods to continue services when the services are unavailable in-person.

The main themes that emerged from our Research Topic are useful to guide policy makers and health/care practitioners in protecting child and parent mental health and promoting child development post-pandemic. The critical need of support for parents was clear in many of the research papers. To address widespread family challenges and needs during the pandemic, some key considerations will be important. First, implementing evidence-based programs that can treat parents' fear, parenting stress, and parents' mental health are crucial. The research suggests a continued focus on parents with depressive symptoms, and methods to promote supportive and sensitive parenting and family resilience. Second, to meet the needs of families most at risk, ensuring low cost, flexible and remote support is needed. Support that considers a variety of online, telephone, or physically distanced service delivery options to accommodate family schedules and comply with physical distancing. Third, novel technologies providing digital delivery of psychological services for families played a crucial role during the pandemic. These new approaches need to become part of our service options post-pandemic, as they allow outreach to a large number of families. More research on the effectiveness of virtual or tele-services designed for families experiencing a range of health, household and psychosocial risk factors, are of crucial importance. Research that examines not only outcomes but the factors around what works best for whom. One aspect of the pandemic is that it has increased our awareness of the devastating impacts of risk factors on parents and children but has also, more positively, allowed us to think in new ways about how we work with families using new technologies while improving access to services and improving outcomes.

## Author Contributions

All authors listed have made a substantial, direct, and intellectual contribution to the work and approved it for publication.

## Funding

This work was partially supported by grant RF-2016-02361884 to RM from Italian Ministry of Health.

## Conflict of Interest

The authors declare that the research was conducted in the absence of any commercial or financial relationships that could be construed as a potential conflict of interest.

## Publisher's Note

All claims expressed in this article are solely those of the authors and do not necessarily represent those of their affiliated organizations, or those of the publisher, the editors and the reviewers. Any product that may be evaluated in this article, or claim that may be made by its manufacturer, is not guaranteed or endorsed by the publisher.
